# Acknowledgment of *Ad Hoc* Reviewers

**DOI:** 10.1128/mSystems.01223-20

**Published:** 2020-12-22

**Authors:** Jack A. Gilbert

**Affiliations:** a University of California, San Diego, San Diego, California, USA

## EDITORIAL

The extraordinary complexity and challenges that faced us all in 2020 fell hardest on those who are traditionally underserved, marginalized, and experiencing prejudice. The unprecedented lockdown reshaped our work environment and changed how we report the results of our scientific investigation, but it also caused huge pressure on certain groups, often disproportionately impacting early-career scientists, women, and minorities. Therefore, as 2020 finally draws to a close and we look to a brighter new year, it is a good time to extend our thanks to all the reviewers who have gone “above and beyond” to ensure the integrity of scientific research reporting in the face of extreme pressure due to isolation, homeschooling, and limited Wi-Fi. Your sacrifice ensures that our truth will not be eroded and that we can still trust research reported in *mSystems*.

Beth M. Adamowicz
Karthikeyan Adhimoolam
Joshua N. Adkins
Matthew Agler
Eric Aguiar
Stephen Dela Ahator
Hajar Fauzan Ahmad
Irfan Ahmad
Warish Ahmed
Palok Aich
Omid Akbari
Begum Alaybeyoglu
Michaeline B. N. Albright
Delphine Aldebert
Gajender Aleti
Nabil-Fareed Alikhan
David Alland
Jacob Allen
Emma Allen-Vercoe
Alexandre Almeida
Lauren Victoria Alteio
John Alverdy
Katherine R. Amato
Carol Viviana Amaya Gomez
Ole Herman Ambur
Jan Amend
Alongkorn Amnuaykanjanasin
Amitesh Anand
Annette Carola Anderson
Gregory G. Anderson
Anand Andiappan
Andres Andrade Dominguez
Nana Y. D. Ankrah
Sten Anslan
Rebecca Ansorge
Haike Antelmann
Vijay C. Antharam
Maciek R. Antoniewicz
André Antunes
L. Caetano M. Antunes
Victor Hugo Aquino
Manuel Aranda
Gabriele Arcari
Catherine R. Armbruster
Zach Armstrong
James T. Arnone
Carol Arnosti
Gunjan Arora
Mario L. Arrieta-Ortiz
Melinda Marie Ashcroft
Marina Elisabeth Aspholm
Laetitia Attaiech
Jennifer M. Auchtung
Fikri Y. Avci
Mian Muhammad Awais
Selcan Aydin
M. Andrea Azcarate-Peril
Oliver Baars
Paul Babitzke
Taeok Bae
Chengrong Bai
Jonathon L. Baker
Paul William Baker
Sreejata Bandopadhyay
Carlos Barajas
Wendy S. Barclay
Mohammed Barigou
Lars Barquist
Rodolphe Barrangou
Frederic J. Barras
Jonathan Barratt
Francois-Xavier Barre
Ryan P. Bartelme
Luther A. Bartelt
Mirko Basen
Christine Marie Bassis
Florian F. Bauer
Anthony D. Baughn
Jose M. Bautista
Sean K. Bay
Jagadeesh Bayry
William N. Beavers
Christine Beemelmanns
Oded Beja
Pedro Belda-Ferre
Lisa K. Belden
Alfonso Benítez-Páez
Sean Benler
Sarah Ben Maamar
Gian Maria Niccolo Benucci
Maureen Berg
Beatrice Berger
Megan Bergkessel
Renaud Berlemont
Emma Bermingham
Anthony Bertagnolli
Andrew David Berti
Katja Bettenbrock
Oliver K. I. Bezuidt
Kyle Bibby
Ethan Bier
Sarah Bigot
Elisabeth M. Bik
Steven Biller
Jordan T. Bird
Kyle Bittinger
Alain Blanchard
Jeffrey Blanchard
Jake Bledsoe
Alissa Bleem
Robert M. Blumenthal
Said Bogatyrev
Nicholas Andrew Bokulich
Jennifer M. Bomberger
Ivo G. Boneca
Carla Yaneth Bonilla
Erika Bonsaglia
Elizabeth Boon
Ricardo M. Borges
Olivier Borkowski
Guillaume Borrel
João Botelho
Michael J. Bouchard
Paul Boudreau
Alice Boulanger
Bob Bourett
Joel Bozue
Günter Brader
Patrick H. Bradley
Kristoffer Brandvold
Christopher Bräsen
Mya Breitbart
Michael R. Brent
Vanessa Brisson
Ilana Lauren Brito
Mark Brönstrup
Amanda Brown
Daniel R. Brown
Sebastian Bruchmann
Harald Bruessow
Sascha Brunke
James Brunner
Jason Bubier
Daniel H. Buckley
Michelle Monique Conni Buckner
Sara Ashley Bumgardner
Catherine M. Burgess
Sara Burgess
Alita R. Burmeister
Jeremy P. Burton
Susheel Bhanu Busi
Lucélia Cabral
Daniel Caddell
Jianfeng Cai
Paolo Calligari
Magdalena Calusinska
Antonio Camargo
Barbara J. Campbell
Hüseyin Can
Xinyun Cao
Andrés M. Caraballo-Rodríguez
Valerie Jean Carabetta
Alessandra Carattoli
Nicholas H. Carbonetti
Jane M. Carlton
Adrian Carper
Tyler J. Carrier
John R. Casey
Santiago Castillo-Ramírez
Duccio Cavalieri
Yunrong Chai
Stephane Chaillou
Kok Gan Chan
Siu H. J. Chan
Christopher Peter Chanway
Yanjie (Jay) Chao
Matthew R. Chapman
Gina M. Chaput
Benoit Chassaing
Jonathan Chekan
Baodong Chen
Casey Chen
Fusheng Chen
Jen-Tsung Chen
Jun Chen
Liang Chen
Stanley H. Chen
Tingtao Chen
Wei Chen
Marc G. Chevrette
Loo Wee Chia
Jason Chin
Byung-Kwan Cho
Sanghyun Cho
Ah Reum Choi
Jessica Chopyk
Maria Chuvochina
Nico J. Claassens
Dennis Claessen
Thomas Clavel
José Francisco Cobo Díaz
Ana Cláudia Coelho
Luis Pedro Coehlo
Aidan Coffey
Natalie Cohen
Devin Coleman-Derr
James Collins
Georg Conrads
Lydia M. Contreras
Tyrrell Conway
Rene Cortese
Paul Cos
Ömer Coskun
Zachary Costliow
Jason Crawford
Alexander Crits-Christoph
Daniel Croll
Sean Crosson
Sean Andrew Crowe
David E. Crowley
Zhongli Cui
Michael Cunliffe
Cameron R. Currie
Johanna Patricia Daily
Benjamin Dainat
Martina Dal Bello
Robert E. Danczak
Washington DaSilva
Maude David
Seana K. Davidson
Shawn Davidson
Thomas Dawson
Allan Degen
Hidde de Jong
Tom Delmont
Jill Dembrowski
Xiangyu Deng
Xin Deng
Yijie Deng
Jonathan J. Dennis
Todd DeSantis
Megan De Ste. Croix
Suzanne Devkota
Travis J. De Wolfe
Jonathan D. D’Gama
Avantika Dhabaria
George C. diCenzo
Gregory J. Dick
Christian Diener
Christina Diez-Vives
Stephen P. Diggle
Yousong Ding
Alden Conlan Dirks
Jocelyne DiRuggiero
Ray Dixon
Ulrich Dobrindt
Daryl Domman
Rodolpho Martin do Prado
Mert Döşkaya
Ryan S. Doster
Nir Drayman
Pavel Drevinek
Alexandre Drouin
George L. Drusano
Melissa Dsouza
Chao Du
Rebbeca Duar
Vikash Kumar Dubey
Daniel Christopher Ducat
Lesley L. Duffy
Tim J. Dumonceaux
Colum Dunne
Sanjucta Dutta
Rachel J. Dutton
Mohammed Dwidar
Tom Edlind
Martinique Lefevre Edwards
Eleonora Egidi
Gisli Einarsson
Damian Ekiert
Mikros Emmanuel
Alexandra Emmons
Stephanie Engel
Melinda Anne Engevik
Tobias Engl
Brendan Epstein
Carmen Escudero-Martinez
Alessandra S. Eustaquio
Sarah Elisabeth Ewald
Jeremiah Faith
Aili Fan
Kunkun Fan
Seamus Fanning
Sara Federici
Conor Feehily
Adam M. Feist
Tamás Felföldi
Fengqin Feng
Jean-Luc Ferat
Jane Ferguson
Pedro Eduardo Ferreira
Elvira Fiallo-Olivé
Daniel Figeys
Celio Fernando Figueiredo Angolini
Gilberto E. Flores
Stelios Fodelianakis
Herrison Fontana
Larry J. Forney
Jane Foster
Leonard J. Foster
Hugo Fraga
Michael T. France
Emanuela Frangipani
Eric A. Franzosa
Lori Frappier
David N. Fredricks
Kelle C. Freel
Iddo Friedberg
Jens Christian Frisvad
Takashi Fujishiro
Tak Fung
Chikara Furusawa
Iwona Gabriel
Ana Cristina Gales
Michael G. Ganzle
Ferran Garcia-Pichel
Julia M. Gauglitz
Patrice Gaurivaud
Jennifer Geddes-Mcalister
Edward Geisinger
Mikhail S. Gelfand
David Gell
Landon John Getz
Benjamin Gewurz
Raad Gharaibeh
Steven R. Gill
Caitlin Gionfriddo
Jorge A. Giron
Anum Glasgow
Bettina Glasl
Sydney Glassman
Erin S. Gloag
Matthew Robert Goddard
Ben Gold
Isabel Gómez-Hurtado
Alicia González
Claire L. Gorrie
Celia W. Goulding
Cédric Grangeteau
Michael Jeffrey Gray
Todd M. Greco
Kacy Greenhalgh
Maureen Groer
Kirsten Grond
Harald R. Gruber-Vodicka
Danxia Gu
Raúl Guantes
María-Eugenia Guazzaroni
François Guérin
Simone Guglielmetti
Sohini Guha
Caitriona M. Guinane
Vadim M. Gumerov
C. J. Guo
Tony Gutierrez
Laila Gutiérrez-Kobeh
Michelle Gwinn-Giglio
Elaine M. Haase
Yang Hai
Henry J. Haiser
Vanessa Hale
Fabien Halkett
Ruth M. Hall
Kimberly H. Halsey
Mohammad Hamidian
Dongsheng Han
Xinyan Han
S. Emilia Hannula
William R. Harcombe
Dayna M. Harhay
Jonathan Harris
Ellie Harrison
Xavier Harrison
Erica M. Hartmann
Caroline S. Harwood
Koji Hase
Roland Hatzenpichler
Alan R. Hauser
Jane Hawkey
Alyse Hawley
Yan He
Jack A. Heinemann
Julia Helmecke
Nicholas C. K. Heng
Michael Henson
Elisabeth A. Herniou
Robert L. Hettich
Steven Higgins
Russell Hill
Deborah M. Hinton
Itaru Hirai
Alec J. Hirsch
Shang-Tse Ho
Laura Hobley
Adam J. Hockenberry
Lucas R. Hoffman
Emily B. Hollister
Patrick Finn Horve
Fuping Hu
Shijia Hu
Yuntao Hu
Hsin-Ho Huang
Jean J. Huang
Zhijian Huang
Luciano F. Huergo
Meredith A. Hullar
Christopher Hunter
Ryan C. Hunter
Yi-Xin Huo
A. Hurley
Filip Husnik
Alan Hutchison
Mathilde Hutin
Robert Hutkins
Edel Maria Hyland
Alex Hynes
Zehra Esra Ilhan
Katherine Ilia
Thomas R. Ioerger
Md. Tofazzal Islam
Emoleila Ejiya Itoandon
Siva Sankari Iyer Mani Sankaran
Jonathan Jacobs
William R. Jacobs
Mario Jacques
Dieter Jahn
Hee-Chang Jang
Paul R. Jaschke
Robert Jenq
Paul R. Jensen
Garilyn Jentarra
Gerdhard L. Jessen
Michael Jewett
Aaron Jex
Wei Jia
Shuo Jiao
Diego Javier Jiménez
Jay-Hyun Jo
Julia Johnke
Abigail J. Johnson
Michael David Leslie Johnson
Timothy J. Johnson
Daniel S. Jones
Hannah Jordt
Peter Jorth
Christine Josenhans
Susan Joseph
Matthieu Jules
Joshua L. Justice
Nadeem O. Kaakoush
Vladimir R. Kaberdin
Mary E. Kable
Teemu Kallonen
Dae-Wook Kang
Ray M. Kaplan
S. L. Rajasekhar Karna
Peter D. Karp
Justin R. Kaspar
Zain Kassam
Shingo Kato
Tomomi Kawai
Sean M. Kearney
Scott T. Kelley
Christina A. Kellogg
Nabla Kennedy
Sander Kersten
Aman Khan
Hemant Khanna
Megan R. Kiedrowski
Patrick Kiefer
Mogens Kilstrup
Jae Su Kim
Young-Mo Kim
Jonathan Klassen
Manuel Kleiner
Stefan Klumpp
Jen Knies
Petr Kohout
Tyler Kokjohn
Carolin Kolmeder
Bethany Kolody
Aleksandra Kolodziejczyk
Yahya Kooch
Janet Cheruiyot Kosgey
Ákos T. Kovács
Rosa Krajmalnik-Brown
Tino Krell
Arjun Krishnan
Andrew M. Kropinski
Jia-Liang Kuang
Jessica Z. Kubicek-Sutherland
Louise Kuhn
Anand Kumar
Ashwani Kumar
Roshan Kumar
Deepak Kumaresan
Ranjith Kumavath
Yakov Kuzyakov
Matthias Labrenz
Christophe Lacroix
Daniel A. Lafontaine
Ariadna Laguna
Leo Lahti
Hsin-Chih Lai
Johanna Lampe
Pawel Laniewski
Rafael Laso-Pérez
Christian L. Lauber
Aonghus Lavelle
Pedro Humberto Lebre
Christopher T. Lefevre
Laura E. Lehtovirta-Morley
Vanessa Leone
Ruth E. Ley
Bailiang Li
Houkai Li
Hui Li
Meng Li
Shiming Li
Xiangrui Li
Yong Li
Zhiyong Li
Chen Liao
Xin-Di Liao
Tami Lieberman
George Liechti
Zachary Liechty
Roland Lill
Shaun Lim
Jonathan Y. Lin
Abigail Lind
Fangqiong Ling
Ning Ling
John J. Lipuma
Sara Little
Guangliang Liu
Guodong Liu
Jinxin Liu
Jun Liu
Long Liu
Wei Liu
Andrew Long
Ana Longo
Allison Lopatkin
Norberto Peporine Lopes
Gabriel L. Lozano
Catherine Lozupone
Xin M. Luo
Andréanne Lupien
Longxian Lv
Michael Lyu
Bin Ma
Yingfei Ma
Roderick I. Mackie
Hannah MacLeod
Karen L. Madsen
Rick M. Maizels
Piotr Majewski
Juraj Majtan
Lucie Anne Malard
Jacob Malone
Serena Manara
Nicasio Mancini
Christopher Mancuso
Rabindra Kumar Mandal
Nathan P. Manes
Ren Mao
Ramona Marasco
Julian R. Marchesi
Maria L. Marco
Abelardo Margolles
Martin G. Marinus
Jeffrey Marlow
Ian P. G. Marshall
Maria Martell
Julia E. Martin
Veronica Martin
Beatriz Martínez
José Luis Martínez
Esteban Martínez-García
Manuel Martinez-Garcia
Maria Elena Martino
Jon Martinson
Vincent G. Martinson
Prince Peter Mathai
Alison Mather
Despoina Mavridou
Xavier Mayali
Melinda Jane Mayer
Chase Mayers
Cameron McBride
Mark J. McBride
John P. McCutcheon
Alice C. McHardy
Aoife J. McHugh
Michael J. McLaren
Lynn M. McMullen
Conor Meehan
Luis C. Mejia
John Scott Meschke
Joseph R. Mihaljevic
Christian Milani
Ian J. Miller
Robert H. Mills
Tanja Mimmo
Kiwamu Minamisawa
Samuel Minot
Biswapriya Biswavas Misra
Caroline Mitchell
Suparna Mitra
Sara Mitri
Jennifer M. Mobberley
Luke A. Moe
Andrew Moeller
Wendy Mok
Babak Momeni
Denise Monack
Jonathan Monk
Kyung Moon
Jamie Morton
Alexander Moschen
Kathy Mou
Quézia Moura
Enock Mpofu
Qinghui Mu
C. S. Mukhopadhyay
Carly Muletz-Wolz
Issa Atanda Muraina
Mario Muscarella
Kevin S. Myers
Carey D. Nadell
Maya Nadimpalli
Ravinder K. Nagpal
Ryohei Thomas Nakano
Jesus Navas-Castillo
Dan Naylor
Evgeniya V. Nazarova
Andrew L. Neal
David M. Needham
Julia Neilson
Roy Neilson
Eric Jorge Nelson
Jiajia Ni
Timothy J. Nice
Martina Niebler
Kang Ning
Ben Niu
Masaru Konishi Nobu
Cecilia Noecker
Michael James Noto
Akos Nyerges
Spencer V. Nyholm
Darren Obbard
Ingrid Obernosterer
Amanda G. Oglesby
Anil Ojha
Anders Omsland
Marc Ongena
Fabini D. Orata
Mehmet A. Orman
Daniel Barros Ortega
Carlos R. Osorio
George O’Toole
Marc Ouellette
Kalon Overholt
Will A. Overholt
Anne-Marie Overstreet
Ben O. Oyserman
Egon Anderson Ozer
Marton Palatinszky
Brian Palenik
Fernando Lucas Palhano
Robert J. Palmer
Bernhard O. Palsson
Kevin Panke-Buisse
Ivan Parra-Izquierdo
Raghuveer Parthasarathy
Laila Partida-Martinez
Sally R. Partridge
Javier Pascual
Edoardo Pasolli
Alessandro Passera
Vinay Pathak
Kiran Raosaheb Patil
Rob Patro
Sushmita Patwardhan
Ian T. Paulsen
Sara Paver
Samuel H. Payne
Talima Pearson
Roger D. Pechous
Christie Peebles
Itsik Pe’er
Yousong Peng
Rita de Cassia Pessotti
Kevin Pethe
William A. Petri, Jr.
Vjacheslav Petrov
Hoang Cong Phan
Caroline C. Philpott
Azul Pinochet-Barros
Gabin Piton
Anna Plantinga
Mircea Podar
Laurent Poirel
Alessandra Polissi
Phillip B. Pope
Welkin H. Pope
Steven L. Porter
Ashok Prasad
Akbar Adjie Pratama
Sarah Pacocha Preheim
Morgan N. Price
John R. Pringle
Sambhawa Priya
Maraike Probst
Lita Proctor
Birgit M. Pruess
Alexandra E. Purdy
Yuanyuan Qu
Robert Andrew Quinn
Ralf Rabus
Marcela Alejandra Radice
Ali Rahnavard
Seth Rakoff-Nahoum
Teppo Rämä
Gayetri Ramachandran
Hazem Ramadan
Vijay Ramakrishnan
Ryan Ranallo
Drauzio Eduardo Naretto Rangel
Simon Rasmussen
Sebastian Rausch
Jayamary Divya Ravichandar
Kasie Raymann
Timothy D. Read
Guislaine Refrégier
Cong Ren
Olaya Rendueles-Garcia
Alejandro Reyes
Sebastián Reyes-Cerpa
Daina L. Ringus
Adam Rivers
Steve Robbins
Leah Roberts
Jennifer Rocca
Dan D. Rockey
Guillermo Rodrigo
Jorge Rodriguez
Mario Alberto Rodríguez
Maria Carolina Rodriguez Daza
Francisco Rodriguez-Valera
Robin Rebecca Rohwer
Fernando Rojo
Kymberleigh A. Romano
Veronica Roman-Reyna
Paola Roncada
David A. Rosen
Daniel E. Ross
Seth Rotz
Hugo Roume
Paul H. Roy
Francesco Rubino
Jakob Ruess
Kelly V. Ruggles
Marc Ruitenberg
Natividad Ruiz
Thomas A. Russo
Feargal J. Ryan
Samuele Sabbatini
Rajib Saha
Jason W. Sahl
Deepak Kumar Saini
Michaela M. Salcher
Howard Salis
Manuel Salmeron-Sanchez
Elisa Salvetti
Ana Sampaio
John C. Samuelson
Álvaro San Millan
Alyson Santoro
Alfonso Santos-Lopez
Christian Santos-Medellín
Rumakanta Sapkota
Martin Sapp
Alain Sarniguet
Guillaume Sarrabayrouse
Yuko Sasaki
Lakkakula Satish
Karin Sauer
Uwe Sauer
Alexei Savchenko
Jimmy H. Saw
Neha Sawhney
Patrick D. Schloss
Stephan Schmitz-Esser
Jochen Schneider
Karin Schnetz
Rebecca A. Schomer
Carla Bianca Schubiger
Monika Stefanie Schütz
Bianca Sclavi
Ninecia Scott
Kimberley D. Seed
Anna Maria Seekatz
Leopoldo Segal
Stephanie Seifert
Shlomo Sela
Aditi Sengupta
Lauren Marie Seyler
Yongqi Shao
Michael Shapira
Gil Sharon
Alaullah Sheikh
Qirong Shen
Zike Sheng
Liat Shenhav
Ahmed A. Shibl
Michael U. Shiloh
William Shoemaker
Francesca L. Short
Steven M. Short
Nachshon Siboni
Jessica R. Sieber
Paul Siegel
Mitchell Singer
Steven Singer
Sonia Singhal
Carolyn M. Slupsky
Younes Smani
Barth F. Smets
Nicholas M. Smith
Sarah Smith
Trever C. Smith
Theo H. M. Smits
Andrea Söllinger
Pacifica Sommers
Mike Seong Son
Utkarsh Sood
Jorg Soppa
Patrick Sorensen
Diana Zita Sousa
Peter Speck
Sanjeeva Srivastava
Eric V. Stabb
Blake W. Stamps
Kenneth M. Stedman
Andrew D. Steen
Ashley A. Stegelmeier
Blaire Steven
Lisa Stinson
Stephen Robert Stockdale
Juergen Stolz
D. G. Storey
Gilles Stouvenakers
Francesco Strati
Wolfgang R. Streit
Giannis Stringlis
Rhona K. Stuart
Xiaoquan Su
Zhengchang Su
Tatsuki Sugi
Muhammad Suleman
Snorre Sulheim
Christopher S. Sullivan
Anne O. Summers
Chaomin Sun
Zheng Sun
Antonet Svircev
Kelly S. Swanson
Marc A. Sze
Éva Szentirmai
Kazuhiro Takemoto
Li Tan
Azuma Taoka
Julien Tap
Suvi Taponen
Florence Tardy
Martin Täubel
Martin Taubert
Leho Tedersoo
Nicolas Terrapon
Herve Tettelin
Bas Teusink
Robert Thänert
Olivier Thas
Casey M. Theriot
Nikhil A. Thomas
Mitchell G. Thompson
J. Cameron Thrash
Ruth E. Timme
Anna D. Tischler
Hirokazu Toju
Magaly Toro
Mónica Torres Beltrán
Moritz Treeck
Laura Treu
Stacey Trevathan-Tackett
Jean-Pierre Trezzi
Anupriya Tripathi
François Trottein
Andrew W. Truman
Renee M. Tsolis
Kieran Tuohy
Tomasz Wojciech Turowski
Jane F. Turton
Shipra Vaishnava
Jesus Valdes
Jake Valenzuela
Lizah Van der Aart
Marjan W. van der Woude
Jordi van Gestel
Marc Warwick Van Goethem
Daria Van Tyne
Jose Vargas-Muñiz
Suzan Pantaroto Vasconcellos
Joachim Vater
Balaji Veeraraghavan
Nic M. Vega
Alain Veilleux
Nadine Veith
Helianthous Verma
Marco A. R. Vinolo
Marie-Joelle Virolle
Emily Vogtmann
Chris Voigt
Martin von Bergen
Lela Vukovic
Joseph Thomas Wade
David M. Wagner
Irene Wagner-Dobler
Jacob Waldbauer
Kevin John Waldron
Elizabeth Walker
Aaron Walsh
David A. Walsh
William A. Walters
Guanghua Wang
Harris Wang
Jianjun Wang
Jinfeng Wang
Jun Wang
Shulei Wang
Sishuo Wang
Wenmei Wang
Xiaoxue Wang
Xueshan Wang
Yan Wang
Zhenyuan Wang
Zhong Wang
Kenneth Wasmund
Karrie A. Weber
Tilmann Weber
Yulong Wei
Rebecca Weiser
Martin Welch
Malcolm F. White
Richard Allen White III
Timothy A. Whitehead
William B. Whitman
Tabor Whitney
Matthias Wietz
Roland Conrad Wilhelm
Peter Williamson
Tomasz Wilmanski
Jennifer R. Wilson
Charles Wimpee
Phitchayapak Wintachai
Jeffrey H. Withey
Patricia G. Wolf
Alan J. Wolfe
Matthew C. Wolfgang
Stephen Woloszynek
Jeffrey A. Woods
Colin Worby
Felipe Christoff Wouters
Kelly C. Wrighton
Fuqing Wu
Weihui Wu
Sander Wuyts
William Wenwei Xiang
Bingbing Xiao
Shichao Xing
Ling Xu
Zhenjiang Zech Xu
Zhi-Xiang Xu
Katherine Xue
Aixin Yan
Dongsoo Yang
Jin-Kui Yang
Jun Yang
Vicky Yao
Min Yap
Laxmi Yeruva
Ryland F. Young
Ying Yu
Natalya Yutin
Livia S. Zaramela
Karsten Zengler
Musu Zha
Kateryna Zhalnina
Haoran Zhang
Jiachao Zhang
Junling Zhang
Quan-Guo Zhang
Ruifu Zhang
Jiangchao Zhao
Kun Zhao
Ni Zhao
Yanlin Zhao
Jusheng Zheng
Jiyong Zhou
Ping Zhou
Shungui Zhou
Wenhao Zhou
Zhemin Zhou
Qiyun Zhu
Yan Zhu
Stefan Zimmermann
Erik R. Zinser
Zhiyong Zong
Jackie Zorz

